# Block iliac bone grafting enhances osseous healing of alveolar reconstruction in older cleft patients: A radiological and histological evaluation

**DOI:** 10.4317/medoral.21991

**Published:** 2018-02-25

**Authors:** Yifei Du, Weina Zhou, Yongchu Pan, Yanchi Tang, Linzhong Wan, Hongbing Jiang

**Affiliations:** 1 Jiangsu Key Laboratory of Oral Diseases, Nanjing Medical University; Nanjing 210029, People’s Republic of China; 2 Department of Oral and Maxillofacial Surgery Affiliated Hospital of Stomatology, Nanjing Medical University, Nanjing, China; 3 Department of Polyclinic dentistry, Affiliated Hospital of Stomatology, Nanjing Medical University, Nanjing, China; 4 Department of Orthodontics, Affiliated Hospital of Stomatology, Nanjing Medical University, Nanjing, China

## Abstract

**Background:**

Older alveolar cleft patients (&12 years old) often have wide bone defect as well as teeth loss, resulting in poor osseous healing with conventional alveolar bone grafting (ABG). In this study, we investigated a surgical technique of block iliac bone grafting for the alveolar cleft reconstruction and evaluated the clinical and radiological outcomes of these cleft patients.

**Material and Methods:**

Fifteen patients were included in this study. All cases received preoperative cone bean computed tomography (CBCT) scans for the alveolar cleft evaluation. Osseous outcomes of block iliac bone grafting were assessed at 1 week, 3- and 6-month postoperatively. Volume changes and bone resorption rates were calculated using the measurement modules of Simplant software. Bone samples from one patient undergoing dental implantation were assessed by micro-CT and histological examination. The morbidities of donor-site were analyzed by clinical examination and questionnaire survey.

**Results:**

The average age of the case series was 18.53±2.50 years. The intraoral incision of thirteen cases healed well. However, two cases had oronasal fistula and graft exposure at 1-week postoperatively. The results of follow-up CBCT scans showed significant resistance to radiation on both sides of the bone graft, suggesting a good osseous healing and new bone formation. The mean residual bone volume was 1.68±0.26 cm3, 1.29±0.23 cm3 and 1.15±0.23 cm3 at 1-week, 3- and 6-month postoperatively. Correspondingly, the mean bone resorption rates in 3- and 6-month postoperative were 21.78±6.88% and 30.66±8.97%, respectively. From micro-CT and HE examinations, the block bone samples exhibited a cancellous structure in which mature bone trabecula and functional blood vessels appeared. The average scores of donor-site morbidities were drastically decreased at 3- and 6-month postoperatively compared with those at 1-week postoperatively.

**Conclusions:**

Our results demonstrated that block iliac bone grafting could achieve satisfying osseous outcomes in older alveolar cleft patients, and this technique provided favorable bony condition for further treatments, especially dental implantation.

** Key words:**Alveolar bone grafting, Block bone grafting, Osseous healing, CBCT.

## Introduction

Alveolar cleft patients need to undergo alveolar bone grafting (ABG) for repairing maxillary defect and providing bony support for tooth eruption. Conventional ABG using autogenous cancellous bone from anterior superior iliac crest is the gold standard, usually combined with orthodontic treatment (1). This surgical-orthodontic protocol is commonly recommended to the alveolar cleft patients of 7 to 11 years old, before the eruption of canine teeth in the period of mixed dentition. In this time period, the lateral incisor and the canine gradually develop into the cleft area (2-5). Therefore, ABG at this age window prevented the erupted teeth from exfoliating into the cleft site. In addition, the erupted teeth could play physiological stimulating effect, which prompted bone remodeling and reduced bone resorption (6,7). However, for older patients who have missed the age window, the results of conventional ABG were controversial and could be fraught with problems of wound dehiscence, graft infection and tooth eruption failure (8,9).

Older patients of permanent dentition often had wider alveolar bone defect due to maxillary growth and tooth loss compared with young patients of deciduous or mixed dentition (9,10). The alveolar defect volume could reach to 5 cm3 in older cleft patients, while the residual bone defect was still 2 cm3 using conventional ABG at 6-month postoperatively (9). The failure of teeth eruption and lack of functional stimulation aggravated the bone resorption of conventional ABG (10). Moreover, the possibility of unhealthy oral habits like smoke may cause poor oral hygiene and increase the risk of bone graft infection in older patients (9,11). Thus, alveolar reconstruction in older cleft patients was a challenge to craniomaxillofacial surgeons.

The effect of ABG on maxillofacial development was uncertain. Some researchers found no significant difference in maxillofacial growth between the cleft children having received ABG and the nongraft controls in general (12). Block bone grafting technique was recommended to patients over 15 years of age when early growth and development of premaxillary region have finished (1). Since this monolithic bone block technique was considered to lack the potential for growth consistent with maxillary development. However, block bone grafting technique has been widely used for bone augmentation in adults, with good long-term outcomes (13,14). The bone block could be trimmed to fit for the alveolar defect (15) or fixed with small titanium plates and screws (16). Recent studies reported on the use of block bone grafting in cleft patients of age window (17,18); however, for older alveolar cleft patients, this technique was rarely reported and the osseous outcomes of bone healing were not well investigated.

 In this study, we conducted a case series of older cleft patients of permanent dentition. Instead of conventional iliac cancellous bone grafting, the block iliac bone grafting with fixation was performed. The osseous outcomes and bone resorption rate were assessed by cone bean computer tomography (CBCT) scans. Bone samples from one patient undergoing dental implantation were analyzed by micro-CT and histological examinations. Donor-site morbidities were assessed by clinical examination and questionnaire survey.

## Material and Methods

-Patients’ selection 

From February 2014 to May 2015, a series of 15 alveolar cleft patients with permanent dentition hospitalized at Centre of Cleft Lip and Palate, Stomatological Hospital affiliated to Nanjing Medical University was selected. All cases were clinically examined and received preoperative CBCT (NewTom VGi, Quantitative Radiology Corporation, Verona, Italy) scans for alveolar defect evaluation. The demographic and clinical data of the enrolled patients were given in  Table 1. The study was approved by the Ethics Committee of the School of Stomatology, Nanjing Medical University, China, and written informed consent was obtained from all patients.

**Table 1:**
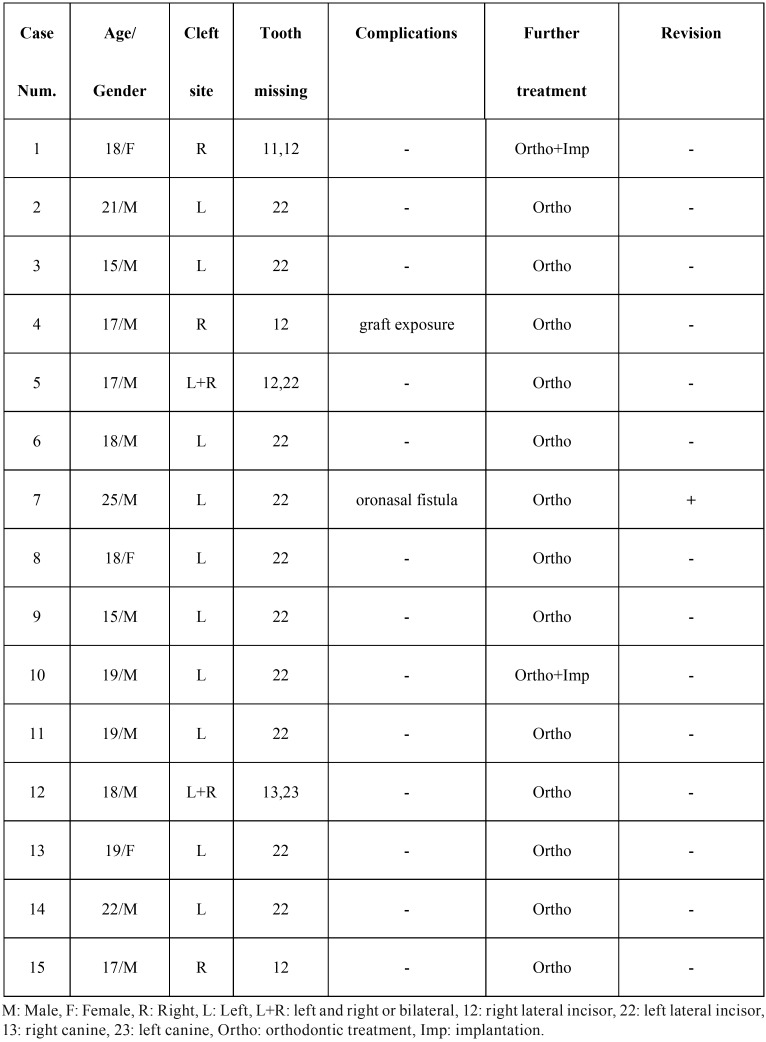
Demographic and clinical data of the enrolled patients.

-Operative technique

Block iliac bone harvesting and trimming 

Block iliac bone harvesting was performed simultaneously with the alveolar preparation by two separate teams. The incision was made laterally to the anterior superior iliac crest. After dissecting iliac crest periosteum, the inner side of iliac crest was exposed carefully to avoid damage to the lateral femoral cutaneous nerve. With 3D reconstruction of the alveolar cleft defect, the prediction of the block bone was determined by preoperative CBCT scans. The osteotomy was performed with Piezosurgery on three sides of the anterior superior iliac crest to create a “block bone”. The final block iliac bone consisted of inner cortical bone and outer cancellous bone. The incision was sutured with layers and the block iliac bone was trimmed by Piezosurgery to fit for the cleft defect.

Block iliac bone grafting and fixation 

**Figure 1:**
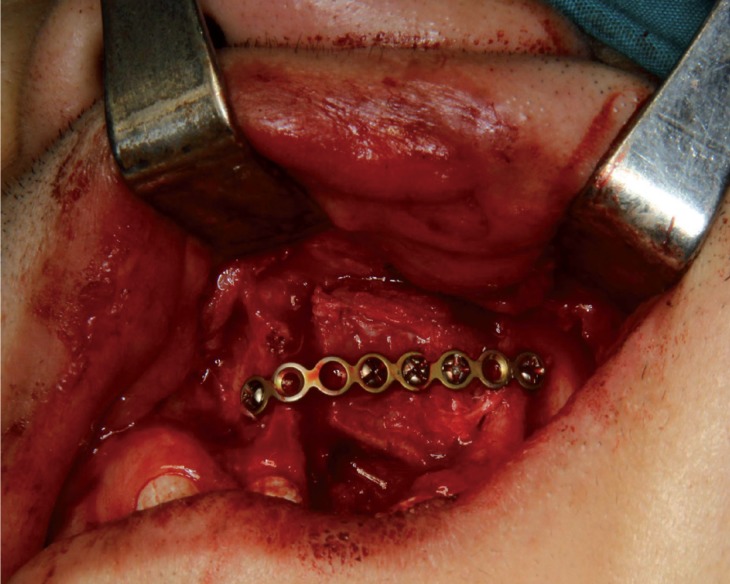
The bone block was fixated to the adjacent maxillae with a long titanium micro-plate.

The alveolar cleft was carefully dissected to accept the bone block. The water-tight suturing of nasal mucosa was particularly important to prevent from the occurrence of oronasal fistula. The trimmed block iliac bone was then placed in the defect with its cortical surface to the buccal side. Micro-plate system (AO, USA) was used to fix block bone to bilateral sides of the defect (Fig. 1). The insufficiency of soft tissue to close the wound was very common and the solution was to separate and loosen the buccal and palatal gingival flaps around the alveolar cleft. The adequate vertical releasing incisions of bilateral gingival were needed, ensuring the closure with no tension.

Outcome analysis

Bone healing and volumetric measurement of block iliac bone graft 

All patients were visually monitored for postoperative complications such as wound infection, oronasal fistula and bone graft exposure by intraoral examinations.

Preoperative and follow-up (1-week, 3- and 6-month) CBCT scans were taken with the same radiation parameters. All data with DICOM format was imported into Simplant Pro 11.04 software (Materialize, Leuven, Belgium) and the coronal, sagittal and horizontal planes of the block iliac bone graft were evaluated. To perform volume rendering, the block iliac bone graft was outlined on each slice using the drawing tools of Simplant software. Collection of all slices with the bone block was stacked to produce a 3D structure and the volume calculation was performed with the same protocol described in previous studies (6,10,14). The volume calculation for each case was repeated three times by the same observer (YC Tang) and the mean data at different follow-up time points was presented with V1 (1-week postoperative), V3 (3-month postoperative) and V6 (6-month postoperative). The resorption rate was calculated as a percentage using the formula (V1–V3 or V6)/V1×100% (6).

Block bone biopsy examination

Bone samples were obtained from case 1 who received two dental implants, with a cylindrical shape of 3.5 mm in diameter and 5 mm in length. The bone samples were immediately sent for micro-CT scans. After that, the bone samples were fixed in 4% parofomaldehyde at 4ºC for 24 hours and decalcified in a solution of 10% ethylenediaminetetraacetic at 4ºC for 2 months. Then, these two samples were dehydrated in a graded series of alcohols and embedded in paraffin. Sections of 4 μm in thickness were prepared for hematoxylin and eosin staining.

Morbidity

The wound healing of iliac crest was examined and any complications such as wound infection, pain and functional limitation were recorded. All patients were investigated by questionnaire to assess the level of postoperative pain, numbness and functional limitations. Standard visual analogue scale (VAS) was used for pain evaluation on a scale of 0 to 10. The numbness was catalogued as four levels (0 = no numbness; 1= mild numbness; 2 = moderate numbness; 3 = serious numbness). For functional limitations, a threepoint grading from 0 to 2 was used (0 = no restriction; 1= restriction with sporting; 2 = restriction with walking). These same surveys were used to measure the donor-site morbidity at follow-up time points postoperatively.

## Results

-General evaluation

The mean age of the patients was 18.53±2.50 years old. Thirteen unilateral alveolar cleft and 2 bilateral alveolar cleft patients were involved. Maxillary lateral incisor was lost in 13 patients, and maxillary canines of both sides were lost in 1 case of bilateral alveolar clefts. One case with right alveolar cleft lost right central and lateral incisors ( Table 1).

-Bone healing and resorption rate

The intraoral wound of all patients was healed well at 1-week postoperatively. However, one case had bone graft exposure at the top of alveolar ridge at 1-month follow-up, which was settled following conservative treatment. Another case with oronasal fistula, resulted in uncontrolled chronic infection of the bone graft. This case was handled for a second surgery to remove the block bone. The unexpected results of these two cases may be due to dehiscence of incision and bone grafts exposure. All patients received orthodontic treatment at 3-month postoperatively, and were recruited to remove the micro-plate system at 6-month postoperatively.

**Figure 2:**
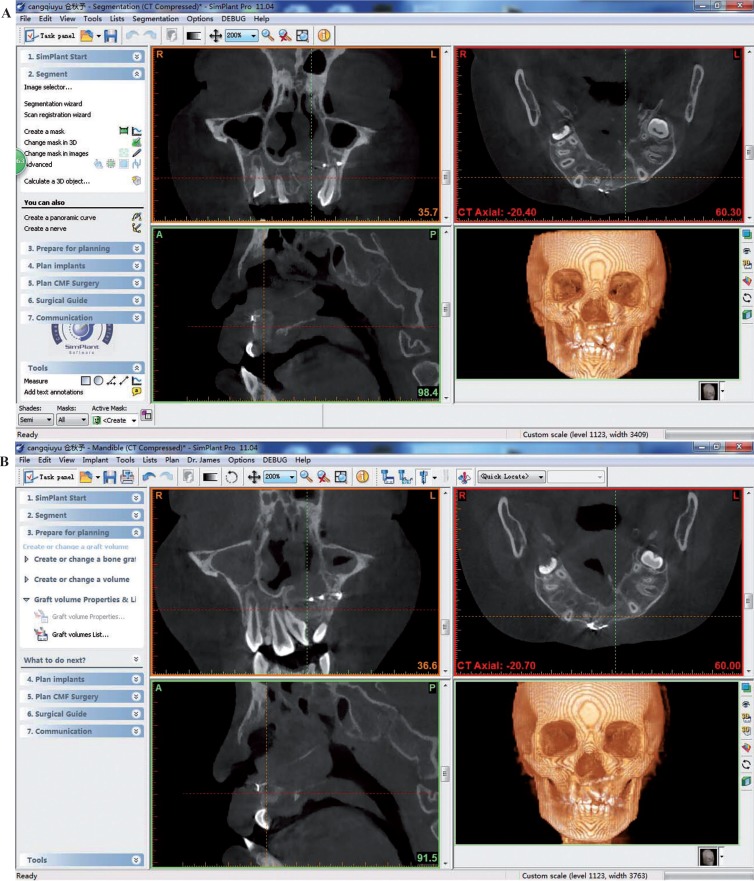
CBCT at 3- and 6-month postoperatively showed new bone formation in the bone graft site. A) The CBCT evaluation at 3-month postoperatively. B) The CBCT evaluation at 6-month postoperatively.

From CBCT scans at 1-week postoperatively, the distinct boundaries between block bone graft and bone defect of alveolar cleft were observed. While the scans at 3- and 6-month postoperatively showed that the boundaries were getting unclear, indicting new bone formation after bone graft procedures (Fig. 2). The mean volume of the block bone grafting at 1-week, 3- and 6-month postoperatively was 1.68±0.26 cm3, 1.29±0.23 cm3 and 1.15±0.23 cm3, respectively. Taking the volume at 1-week postoperatively as baseline reference, the mean resorption rate at 3- and 6-month postoperatively was 21.78±6.88% and 30.66±8.97% ( Table 2).

**Table 2:**
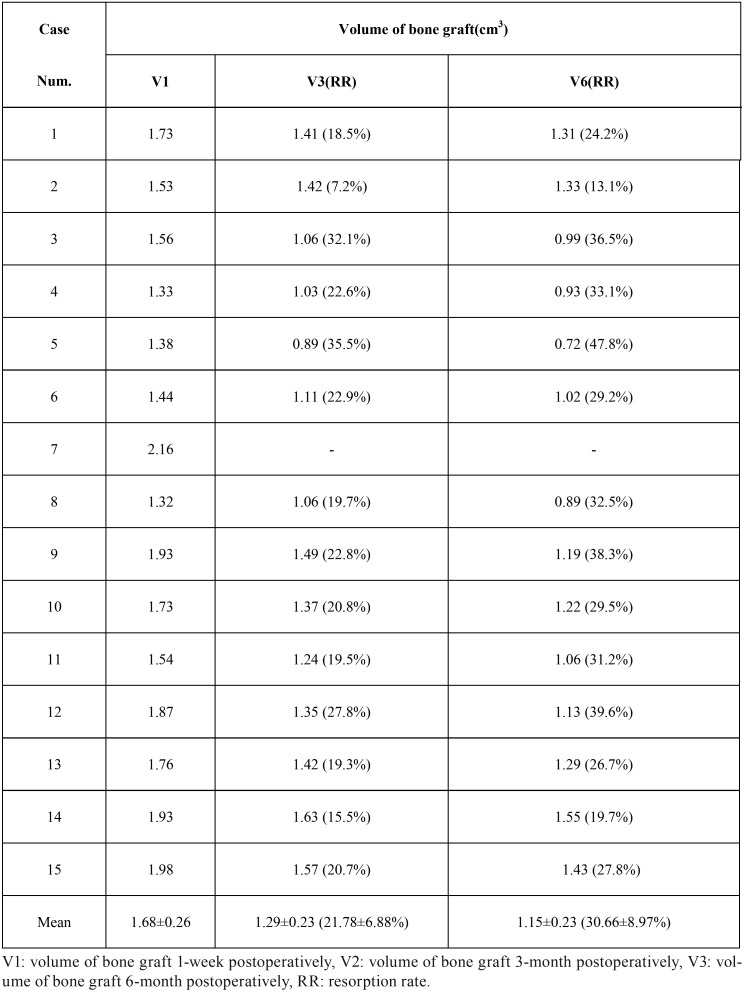
Volumetric calculation of bone graft at 1-week, 3- and 6-month postoperatively.

-Block bone samples examination

**Figure 3:**
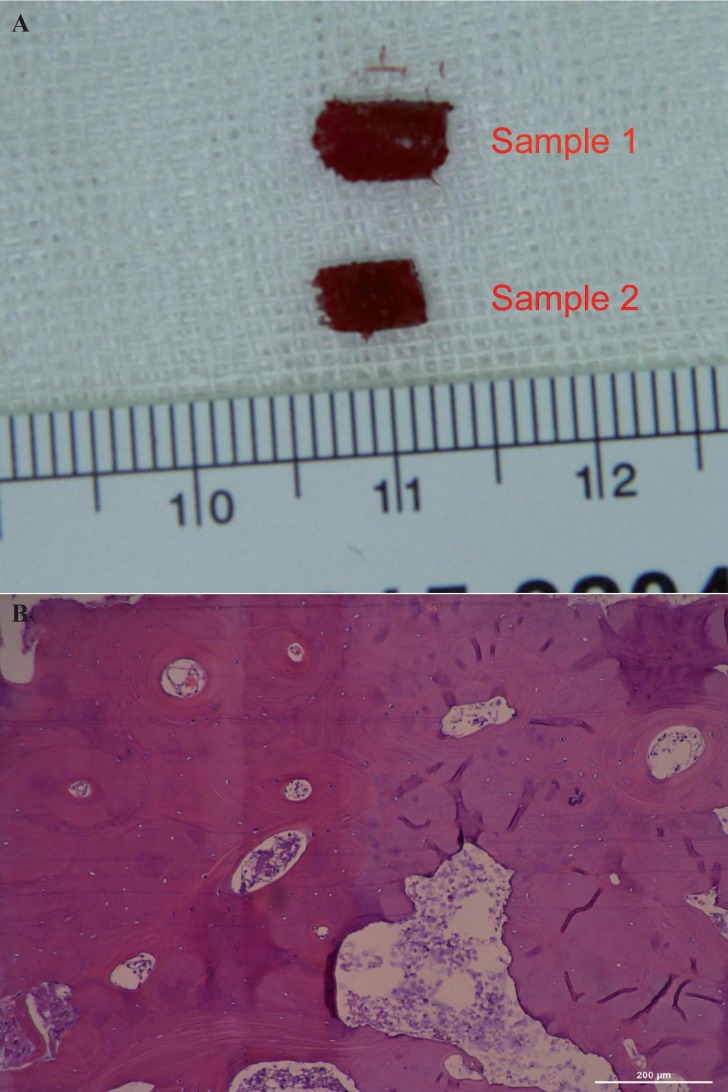
The bone samples of block iliac bone grafting. A) The general observation of the two bone samples from case 1. B) The hematoxylin and eosin-stained examination of bone samples (viewed at 200× magnification).

The block bone samples exhibited a cancellous structure similar to ileum from general observation, which was further verified by micro-CT scans. The 3D reconstruction of micro-CT scans showed a porous form, containing multiple trabecula-like structures. Through hematoxylin and eosin staining, many functional blood vessels were concurrently found in the centre of the calcium deposition. Mature bone formation was observed in a well-proportioned distribution among the porous structures (Fig. 3).

-Morbidity

All patients had excellent healing of the iliac crest wound at 1-week postoperatively without any infection or bleeding. The mean pain scores were 6.47±1.67, 1.73±0.70 and 0 at 1-week, 3- and 6-month postoperatively, respectively. The follow-up results at 3-month postoperatively showed 6 patients suffered from mild numbness and 3 patients from functional restriction of sporting. However, no patient complained numbness or functional limitation at 6-month postoperatively ( Table 3).

**Table 3:**
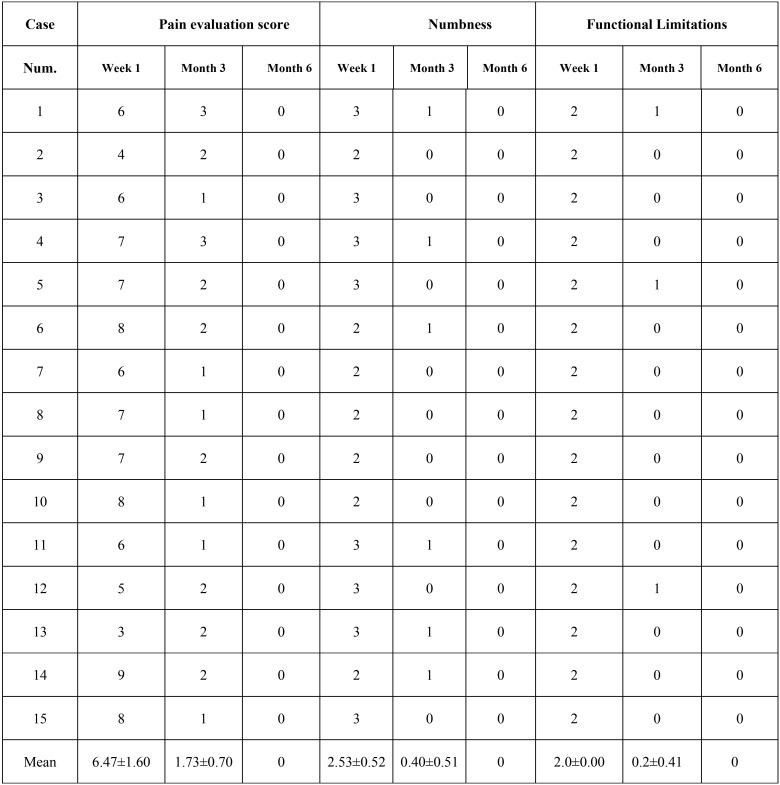
Iliac morbidity investigation of postoperative pain, numbness and functional limitations at 1-week, 3- and 6-month postoperatively.

## Discussion

The surgical-orthodontic procedure for alveolar cleft patient is usually performed between 7 and 11 years of age, consisting of ABG surgery and peri-operative orthodontic treatment. However, some patients miss this age window but need ABG treatment in a delayed fashion. For these older cleft patients, the conditions of the cleft site and oral hygiene are more complicated than that of younger patients. In our case series, all patients were over 15 years old and had one or more teeth loss, predominantly in the location of lateral incisor. Meanwhile, the mean volume of block bone grafting at 1-week postoperatively was 1.68±0.26 cm3 ( Table 2), but the actual volume of alveolar defect could be more due to deficiencies of bone filling. The previous studies including ours, demonstrated that the volume of the cleft defect in younger patients was about 1.0 cm3 based on CBCT or CT scans (10,19-21). In older cleft patients, the volume of alveolar defect could reach to 5 cm3 (9). In addition, the absence of permanent eruption could exacerbate the resorption of cancellous bone grafting, with bone resorption rate up to 36.6% at 6-month postoperatively. While the data was 10.4% if the permanent tooth erupted spontaneously into the bone graft field (6).These results indicated that the tooth loss and skeletal growth could expand the alveolar defect and may impair the osseous outcomes of conventional ABG treatment.

The donor site of block bone could be iliac crest or mandible (16,17). Mandibular bone block could be less resorpted than that from iliac crest for it matched with the embryologic origin on craniofacial bone formation (22,23). However, we preferred bone block from iliac crest rather than from genial or symphysis region, for it could provide adequate bone amount for our cases of wide cleft defect. After preparation of the cleft site, it was necessary to shape the bone block to fit for alveolar defect. Based on our experience, we considered preoperative three-dimensional evaluation of the cleft site and an appropriate bone shaping instrument as two important factors. For the former, CBCT could provide statistical information such as structure and size of the alveolar defect (24). For the latter, we recommend shaping the bones by Piezosurgery, which achieved a high degree of bone cutting efficiency with minimal damage (25).

The block bone was fixed with micro-plate system to ensure a necessary stability of bone healing condition. In the follow-up examination of CBCT, the block bone graft and adjacent alveolar bone were fully integrated completely at 6-month postoperatively. The boundaries between the bone graft and alveolar cleft could not be identified, indicating the new bone formation in the cleft area. Micro-CT and histological findings of bone samples from case 1 also showed excellent mature bone structures. Interestingly, the block bone samples were rich in blood supply and had many functional vessels in the porous structure. This might provide some hints that a good revascularization of block iliac bone grafts contributed to nearly 70% of bone graft retention and new bone formation in 6 months after surgery. For angiogenesis of graft area was curial to bone healing and osseous integration (26). However, limited to the numbers of bone samples, firm conclusion was difficult to draw in the present study. More samples and further animal experiment were needed to elucidate the response of autogenesis ectopic block bone grafting. Base on current results, we only proved that the block iliac bone grafting could be an alternative for older alveolar cleft patients.

Many studies judged the bone graft outcomes by restored marginal bone level percentages using the Bergland scale or Enemark grading system, usually by retroalveolar or panorex radiographs (27-29). However, this two-dimensional bone volume evaluation was considered to greatly overestimate the osseous results of bone grafting. Garib et al. found that the cancellous bone grafting became a thin bone plate on the teeth adjacent to cleft by CBCT scans, although the level of alveolar bone crest was shown to be normal in panorex films (30). These results highlighted the importance of selecting appropriate radiological methods and volumetric assessment programs. Previous studies had calculated the volume and bone resorption rate with same protocols (6,10). In this study, we analyzed the volume changes of block bone grafting by CBCT scanning. The mean bone resorption rates at 3- and 6-month postoperatively were 21.78±6.88% and 30.66±8.97%, respectively. These data were similar to the bone grafts in cleft patients of deciduous or mixed dentition (6,21).

Although the success rate in cleft patients of advancing age decreased, the incidence of complications in the donor/receipt site of older cleft patients was not significantly different from that of young cleft patients (1,11,18). In our series, all patients experienced significant pain and walking dysfunction at 1-week postoperatively. While at 3-month postoperatively, the average scores of pain, numbness and functional limitation dramatically decreased. At 6-month postoperatively, all patients recovered with satisfactory results. These data suggested that the injury of block iliac bone harvesting was relative low in older cleft patients. Additionally, 2 of 15 cases had intraoral complications such as bone graft exposure and oronasal fistula. The incidence was higher than that in previous studies (31,32), reminding us of the importance of extensive separation of the gingival flaps around alveolar cleft.

The shortcomings of this study were its relatively small patient cohort, short-term follow-up and lack of control group using conventional iliac cancellous bone grafting. Compared with pediatric cleft patients, the selection bias of skeleton matured patients resulted in fewer patient samples. The 6- month follow-up was too short to analyze the final osseous outcomes of bone grafting, although the evaluation period for bone resorption rate was less than 1year in the previous studies (6,9).

## Conclusions

The procedure of block iliac bone grafting with reliable fixation led to favorable osseous results in older cleft patients for alveolar reconstruction. It could provide sufficient bone amount for further dental implantation and orthodontic treatment.
